# Historical Perspective: S. Leonard Syme's influence on the development of social epidemiology and where we go from there

**DOI:** 10.1186/1742-5573-2-3

**Published:** 2005-05-25

**Authors:** Irene H Yen

**Affiliations:** 1 University of California, San Francisco, USA

## Abstract

This article accompanies Len Syme's "Historical Perspective: The social determinants of disease – some roots of the movement." It describes some of Len's role in the development of social epidemiology through mentoring investigators and influencing training programs. It also discusses some challenges for the field and ways to move forward.

## Introduction

In "Historical Perspective: The social determinants of disease – some roots of the movement," Professor S. Leonard (Len) Syme describes his professional activities as they related to the emergence of research on the social determinants of health within the field of epidemiology [1]. He writes in some detail about his work at the U.S. National Institutes of Health (NIH) starting shortly after completing his doctorate in medical sociology through to about 1980, by which time he had been a professor at the School of Public Health of the University of California (UC), Berkeley for over a decade.

In this historical perspective, I want to highlight Len's contributions to the field of social epidemiology, with a particular focus on mentoring and training programs. I focus on training because of Len's enormous contribution as a teacher and mentor. Because there are so many of us who have benefited from Len's mentoring, I feel it was important to include other voices and have solicited reflections from colleagues, advisees, and other epidemiologists who are familiar with Len's scholarship. Social epidemiology is maturing as a discipline and there is more work to be done. I thought this essay would be a good place to reflect on some challenges that we face and what we might do to overcome them.

## Analysis

### Research on social determinants – cultivating investigators

I frequently hear people marvel on what an impressive list of names marks Len's career as a professor, both in the quality of the work they are pursuing and the sheer quantity of people. Nancy Adler, professor of medical psychology at UC San Francisco and director of the MacArthur Research Network on Socioeconomic Status and Health, travels all around the U.S. attending meetings on the social determinants of health and health disparities. She told me, "Everywhere I go, when people go around the table to introduce themselves and say how they got there, there are always *several *people in the room who say, 'I'm here because Len Syme was my mentor.' Len is like the universal blood donor." (Nancy Adler, personal communication, March 8, 2005.)

During the period of the primary focus of Len's essay (1968–1980), he advised Lisa Berkman (chair of the Department of Society, Human Development, and Health, Harvard School of Public Health), Robert Hiatt (Director of Population Sciences, UCSF Comprehensive Cancer Center), George Kaplan (Director, Center for Social Epidemiology and Population Health, University of Michigan), and Michael Marmot (Professor of Epidemiology, University College London and Chair of the World Health Organization Commission on the Social Determinants of Health) among others. Since 1980, he was the major thesis advisor to Jack Guralnik, Mary Haan, Nancy Krieger, John Lynch, Teresa Seeman, Marilyn Winkleby, and many others who are doing important work, though perhaps not in social epidemiology nor necessarily in an academic setting.

I met Len in 1988 when I entered the master's degree program in epidemiology and biostatistics at UC Berkeley School of Public Health. I was a member of the third class of students admitted for a two-year master's degree program. Prior to 1986, Berkeley had only offered a one-year master's degree curriculum in epidemiology for people who had prior graduate training, mostly medical doctors. For the two-year program, we were required to attend a core seminar. The core seminar gave us an overview of epidemiology as a discipline (including historical readings), key concepts, a chance to learn about individual faculty research emphasis, and a place for discussion. The faculty in charge rotated; our year, it was Len's turn. I completed the master's degree and took a job with the Centers for Disease Control and Prevention.

I returned to Berkeley in 1993 to pursue a doctorate. Len had already been a strong influence on my thinking as a budding epidemiologist having been the lead faculty for the core seminar during the master's degree program. Len introduced key epidemiological concepts to me, such as a basic definition of epidemiology, "epidemiology is the study of the distribution of diseases in populations." As he had been doing since he started teaching epidemiology, he had all of us read Durkheim and vividly emphasized Durkheim's contribution. I can still hear Len's voice highlighting Durkheim's work, showing the *most *personal of all acts (suicide) varied by region over a period of several decades. Len would stress the significance of the finding by asking provocative questions. How could the rates be so stable over time since clearly the people involved were different? Always an accomplished story teller, Len would unfold the mystery explaining how Durkheim had found that these regions differed by dominant religion, the Catholic regions had different rates of suicide from the Protestant. Ultimately Durkheim proposed the concept of *anomie*. Alongside Durkheim's work, Len talked about the futility of behavioral interventions, pointing out that if we only target behaviors (e.g. smoking, exercise, diet) without targeting root causes, we will have to repeat our work in cohort after cohort of populations. These sorts of stories led me to want to focus on the root factors that underlie the behaviors and eventually I chose to focus on neighborhood environment. Len was my major dissertation advisor.

Len's students appreciate his personalized attention, challenging intellectual arguments, and ongoing support regardless of professional choices. Before taking his current position at UCSF, Robert Hiatt was Deputy Director, Division of Cancer Control & Population Sciences, National Cancer Institute. He reflects, "In an unplanned way Len has been a critical influence on me throughout my career. First, by introducing me to psychosocial perspectives in epidemiology while in training, then by directing me into community intervention research, and finally through indirect, but very real, ways by influencing my work at the National Cancer Institute where I generated research programs in population health." (Robert Hiatt, personal communication, 2005). Here is another reflection from Nancy Krieger, Department of Society, Human Development, and Health, Harvard School of Public Health:

Len Syme was and continues to be a critical mentor for me, in the very best sense of the term. Len consistently has encouraged critical thought and fresh examination of long-standing problems of social inequalities in health. He likewise has always emphasized the relevance of social analysis for understanding public health problems, recognizing that investigating population health requires population level theories. The challenge thus becomes integrating population level social and biological thinking, and Len never once suggested to me this was a waste of time! In fact, when he was my doctoral advisor, he supported strongly my early work on thinking through the intertwined social and biological determinants of breast cancer across the lifecourse, just as he also supported strongly my work on using theories of social class to inform measurement of social inequalities in health and theories of race relation to inform research on how racism harms health. To Len, epidemiology was first and foremost a science of understanding the population distributions of disease, not simply a set of methods to apply to quantitative data that happened to be about health – and it is to his credit that this orientation not only is strong but growing among the many he has taught and influenced. (Nancy Krieger, personal communication, May 4, 2005)

From Lisa Berkman, Chair of the Department of Society, Human Development, and Health, Harvard School of Public Health:

At the time I was thinking about my dissertation, there were only a few studies out there about how social conditions might influence health. The data from Japan was of course very influential. At the time I was also studying social networks in the sociology department with Claude Fisher. Len's perspective was invaluable. He has always encouraged his students to follow their noses and be critical thinkers. His mentoring was invaluable, he questioned and questioned until I finally had a coherent story to tell. (Lisa Berkman, personal communication, May 9, 2005)

### Research on social determinants – Explicit training programs

My classmates in the master's degree program at Berkeley noticed that none of the professors in the epidemiology portion of our program had doctoral degrees in epidemiology, but rather were trained in other disciplines, primarily medicine. Our cohort's disciplinary pasts were even more varied including people who had majored in history, creative writing, environmental studies, political science, and anthropology. I believe that the influx into epidemiology of people from outside of medicine has contributed to the widespread interest in non-clinical non-behavioral factors such as discrimination, social capital, and income inequality. This increased interest is leading to formal training programs in the social determinants of health or population health.

Beginning in 1991, the Harvard School of Public Health established the Health and Social Behavior Department. (In 2003, this Department was re-organized together with the Maternal and Child Health department into the department of Society, Human Development, and Health.) At the time, it was one of the first of its kind, with a stated mission "to identify the social and behavioral determinants of health and to develop and evaluate interventions and policies leading to the improvement of the public's health and quality of life"[2].

More social determinants or population health masters and doctoral training programs are emerging. UC Berkeley began a Health and Social Behavior Masters program in 1999. The University of South Florida approved a socio-health sciences MPH in 2004. Also in 2004, University College London established a MSc program in Health and Society: Social Epidemiology.

Multi-discipline approaches to research have been in vogue of late. Len mentions the Robert Wood Johnson Health and Society Scholars (HSS) postdoctoral training program. HSS had its first cohort of scholars in 2003. Pamela Russo, senior program officer in the Health Group of the Robert Wood Johnson (RWJ) Foundation, credits Len with laying the groundwork for the creation of such a program:

I would say that Len'sthinking, mentoring, and approach were seminal in the evolution of transforming the older narrow medical and biological paradigm for epidemiology to a much broader range of determinants of health captured in the population health approach. ... He personally influenced or mentored many of the current H&SS faculty and site directors – including Lisa Berkman, George Kaplan and others. This program would not have developed if not for Len and his mentees having built the foundations of the field. He broadened the horizon of what influences health status. He also has been a powerful champion of research and interventions to reduce health disparities between populations throughout his career. (Pamela Russo, personal communication, March 11, 2005)

Readers may also be familiar with the Kellogg Scholars in Health Disparities which began in 2001 and includes a focus on social determinants. The director of this program, Barbara Krimgold, similarly recognizes Len's strong influence on the structure, emphasis of multiple disciplines, and inclusion of policy perspectives in the program (Barbara Krimgold, personal communication, March 16, 2005). Len continues mentoring fellows in the RWJ HSS and Kellogg programs who are at the UC Berkeley/UC San Francisco joint training sites.

### Research on social determinants – institutional structures

As mentioned above, in his teaching Len consistently emphasizes the population health and prevention themes to public health and the concept that epidemiology was supposed to be the core scientific method serving public health. He stresses that even though epidemiology rhetoric was about investigating ways to prevent disease, more often the field was constrained by the medical model of dividing the body into organ systems. Looking at NIH during the period that Len's essay focuses on (1968–1980), one can see that the institutes were predominantly concerned with parts of the body or diseases. For example, the National Heart, Lung, and Blood Institute, National Institute of Diabetes & Digestive & Kidney Diseases, National Cancer Institute, and the National Institute of Allergy and Infectious Diseases are four of the six institutes established in the 1940s. The Office of Behavioral and Social Sciences Research (OBSSR) was established in 1995. The first director of the OBSSR was Dr. Norman Anderson. Dr. Anderson describes some of Len's more recent influence on the NIH research agenda:

Len's research, and that which followed it, was the basis of the first ever NIH-wide conference on social determinants of health and illness, entitled, "Toward Higher Levels of Analysis: Promise and Progress on Social Aspects of Illness", which was sponsored by OBSSR. The conference led to a research agenda for the funding of social science research at NIH. So the foundation that Len created was critical to the infusion of a social science perspective at NIH. (Norman Anderson, personal communication, March 15, 2005)

The conference that Dr. Anderson refers to was held in 2000, the same year that the National Center for Minority Health and Health Disparities was established.

U.S. funding opportunities for research on the social determinants of health are heavily influenced by the NIH agenda. In 1999, the National Institute for Environment Health Sciences (NIEHS) spear-headed research on health disparities, first by hosting a series of workshops around the country which academic researchers, government agency staff, and community organization staff attended to discuss priorities. One result was the November 1999 release of the RFA, "Health disparities: linking biological and behavioral mechanisms with social and physical environments." According to Dr. Frederick Tyson, one of the organizers of the workshops and authors of the RFA, Len's "work on how socioeconomic factors influence health outcomes is a major foundation to the RFA." (Tyson, personal communication, March 10, 2005).

Len has also been influential outside the US. In the mid 1980s, Dr. Fraser Mustard (former Vice President of the Faculty of Health Sciences of McMaster University and first President of the Canadian Institute of Advanced Research (CIAR)) was investigating the underlying causes of heart disease in order to prevent it. He was referred to Len because of the MRFIT Study. Apparently, Len argued that not enough was known about the causes to prevent heart disease. Their discussions led Dr. Mustard to invite Len to serve on the advisory committee of the CIAR Population Health Program, which started in 1987. (John Frank, personal communication, March 24, 2005). In part, as an outgrowth of the work of the Population Health Program, in 2001, The Canadian Institutes of Health Research, the Canadian equivalent of the U.S. National Institutes of Health, established the Institute of Population and Public Health (IPPH). Len is a member of the IPPH advisory board and according to the scientific director of the IPPH, during these four years, "he has materially advanced the Institute's development by his unwavering commitment to excellence in research, and his tenacious support for bold investments that can 'make a difference' in building the field of population and public health research in Canada." (John Frank, personal communication, March 21, 2005).

### Research on social determinants – general influence on the field

Aside from directly influencing the training and funding programs mentioned above, social epidemiology was strongly influenced by Len's research. Bruce Dohrenwend, Professor of Epidemiology at Columbia Mailman School of Public Health, reacts to Len's essay:

He was so modest in describing his own achievements and so generous with his praise of others that his own major contributions might be missed by a novice reader. One of these, especially, should be highlighted. This is his leadership role in investigating a series of imaginative hypotheses developed to explain the stunning finding that Japanese men who had migrated to California had rates of coronary heart disease that were five times higher than in their counterparts in Japan. This work is a major achievement, and Len was at the center of it. (Bruce P. Dohrenwend, personal communication, March 28, 2005.)

Not surprisingly, Len's work has reached far. Töres Theorell, director of the National Institute for Psychosocial Factors and Health and Professor of Psychosocial Medicine at the Karolinska Institute in Stockholm, Sweden wrote:

I am of course not the only one here who has been influenced by Len Syme. In particular, social support was an extremely important subject for several researchers here, for instance Per Olof Ostergen in Malmo and Kristina Orth Gomer in Stockholm. So if you look at the reference list of these people, you will find Len Syme's name. Len Syme wrote about our book *Healthy Work *(Karasek and Theorell 1990) in several places and has been driving the idea that 'control in life' would be an important research dimension. ... I am a great admirer of Len, whose constructive ideas and engaged helpfulness has always been a star. (Töres Theorell, personal communication, March 26, 2005.)

## Conclusion

At the end of Len's essay, he reflects that while a tremendous amount of progress has been made, the advances have been met with opposition and suspicion. As someone who is trying to participate and make contributions in the field of social epidemiology, I can attest to the resistance and skepticism. In fact, the idea of "social epidemiology" as a subfield was recently debated in a set of commentaries in the *International Journal of Epidemiology *[3-5].

There are two steps that we can take to build the credibility and contributions of social epidemiology. One of them Len brings up in his essay, the pressing need to bring social theory into the picture. Social theory should be a necessary piece of the conceptualization of research questions by more people who are engaged in research on social factors or population health and more fully incorporated into our training programs.

The need for deeper understanding and application of social theory has already been taken up by some people in the field[4-9]. Others have made strong arguments for the importance of incorporating theory into public health practice more broadly[10,11]. At the risk of oversimplifying, here is a brief overview of the crux of these arguments: Investigating the social determinants of health has turned our attention to the role of income or wealth, race/ethnicity, and education. Instead of including these variables in our multivariate models as covariates, they could be the central variables. Income/wealth, race/ethnicity, and education are the product of social processes and their meanings cannot be measured simply by individual self-report. Social theories provide containers or frameworks with which to understand the social processes and hypothesize about how and why they are relevant to health. A basic analytic theme in social theory is the structure-agency duality[12]. Structures are social institutions such as the family, political institutions, and economic relations. Agency refers to an individual's capacity to act deliberately or to exercise power[13]. Social theories explain how the tension between structures and agency play out. Incorporating and applying theories can inform and strengthen our research questions.

The second step that is tied into the incorporation of social theory is the need for social epidemiology to have a practice-based anchor. In other words, we need grounding in everyday activities. In this way, the field would gain a sorely needed practice perspective and the accompanying legitimacy and credibility. Recent discussions which press for more participatory research are one means to bring in this element [5]. By including the voices of the people who are most directly affected by the processes under study and by holding close the end goal of designing programs, the research will gain more solid grounding. Another way to think about the practice-research link for social epidemiologists is to look to colleagues in other subfields within epidemiology. For example, clinical epidemiologists work closely with physicians and nurses. Their research findings can directly contribute to clinical practice. Clearly both sides are enriched by the contributions of the other. Social epidemiology as a discipline must establish formal ties to practice-based fields, such as city and regional planning, public policy, and education, in order to inform and be informed.

Social epidemiology owes Len Syme a lot. We can repay him by asking tough questions, holding ourselves to high standards, and serving as mentors.

## Abbreviations

CIAR – Canadian Institute of Advanced Research

HSS – Health and Society Scholars

MPH – Masters in Public Health

MRFIT – Multiple Risk Factor Intervention Trial

MSc – Masters in Sciences

NIH – National Institutes of Health

OBSSR – Office of Behavioral and Social Sciences Research

RFA – Request for Applications

RWJ – Robert Wood Johnson

UC – University of California

UCSF – University of California, San Francisco

## References

[B1] Syme SL (2005). Historical perspective: The social determinants of disease – some roots of the movement. Epidemiologic Perspectives & Innovations.

[B2] http://www.hsph.harvard.edu/Academics/hsb/.

[B3] Zielhuis GA, Kiemeney LALM (2001). Social epidemiology? No way. Int J Epidemiol.

[B4] Krieger N (2001). Commentary: Society, biology and the logic of social epidemiology. Int J Epidemiol.

[B5] Macdonald KI (2001). Commentary: Social epidemiology. A way?. Int J Epidemiol.

[B6] Krieger N (2001). Theories for social epidemiology in the 21^st ^century: an eco-social perspective. Int J Epidemiol.

[B7] Frohlich KL, Corin E, Potvin (2001). A theoretical proposal for the relationship between context and disease. Sociology of Health & Illness.

[B8] Muntañer C (1999). Invited commentary: Social mechanisms, race, and social epidemiology. Amer J Epidemiol.

[B9] Cooper RS, Kaufman JS (1999). Is there an absence of theory in social epidemiology? The authors respond to Muntañer. Amer J Epidemiol.

[B10] Frohlich KL, Mykhalovsky E, Miller F, Daniel M (2004). Advancing the population health agenda: Encouraging the integration of social theory into population health research and practice. Can J Public Health.

[B11] Potvin L, Gendron S, Bilodeau A, Chabot P (2005). Integrating social theory into public health practice. Am J Pub Health.

[B12] Szeter S, Woolcock M (2004). Rejoinder: Crafting rigorous and relevant social theory for public health policy. Int J Epidemiol.

[B13] Giddens A (1979). Central Problems in Social Theory.

[B14] Leung MW, Yen IH, Minkler M (2004). Community based participatory research: a promising approach for increasing epidemiology's relevance in the 21st century. Int J Epidemiol.

